# Preoperative CT and survival data for patients undergoing resection of colorectal liver metastases

**DOI:** 10.1038/s41597-024-02981-2

**Published:** 2024-02-06

**Authors:** Amber L. Simpson, Jacob Peoples, John M. Creasy, Gabor Fichtinger, Natalie Gangai, Krishna N. Keshavamurthy, Andras Lasso, Jinru Shia, Michael I. D’Angelica, Richard K. G. Do

**Affiliations:** 1 School of Computing, Queen’s University, Kingston, Ontario Canada; 2 Department of Biomedical and Molecular Sciences, Queen’s University, Kingston, Ontario Canada; 3 The Oregon Clinic, Portland, OR USA; 4 Department of Radiology, Memorial Sloan Kettering Cancer Center, New York, NY USA; 5 Department of Pathology, Memorial Sloan Kettering Cancer Center, New York, NY USA; 6 Hepatopancreatobiliary Service, Department of Surgery, Memorial Sloan Kettering Cancer Center, New York, NY USA

**Keywords:** Biomarkers, Cancer imaging, Metastasis, Tumour heterogeneity

## Abstract

The liver is a common site for the development of metastases in colorectal cancer. Treatment selection for patients with colorectal liver metastases (CRLM) is difficult; although hepatic resection will cure a minority of CRLM patients, recurrence is common. Reliable preoperative prediction of recurrence could therefore be a valuable tool for physicians in selecting the best candidates for hepatic resection in the treatment of CRLM. It has been hypothesized that evidence for recurrence could be found via quantitative image analysis on preoperative CT imaging of the future liver remnant before resection. To investigate this hypothesis, we have collected preoperative hepatic CT scans, clinicopathologic data, and recurrence/survival data, from a large, single-institution series of patients (n = 197) who underwent hepatic resection of CRLM. For each patient, we also created segmentations of the liver, vessels, tumors, and future liver remnant. The largest of its kind, this dataset is a resource that may aid in the development of quantitative imaging biomarkers and machine learning models for the prediction of post-resection hepatic recurrence of CRLM.

## Background & Summary

Colorectal cancer is the third most common malignancy in the United States with 140,000 new cases annually^1^. Prognosis and treatment depends on the stage of disease, a classification system that takes into account depth of invasion into the bowel wall, spread to abdominal lymph nodes, and presence of distant metastases^2^. Following colonoscopy and diagnosis, contrast-enhanced CT scan of the chest, abdomen, and pelvis is used to evaluate for disseminated metastatic disease. Over 20% of patients will have colorectal liver metastases (CRLM) at presentation, and in those who develop metastases following resection of the colonic primary, the liver represents the most common site^3,4^. In selected patients with liver-predominant metastases, hepatic resection of CRLM is the treatment of choice and associated with a 20% chance of cure^5^. However, the majority of those patients will recur in the remnant liver, so identification and selection of patients likely to benefit most from surgery remains challenging^6^.

During surgical evaluation, CT scans are used to determine feasibility and operative plan. Resection of all hepatic tumors must be accomplished with adequate future liver remnant (FLR) for liver regeneration. These pre-operative images potentially hold data that can help improve the selection of treatments for patients with CRLM. Radiomics is an emerging field in which medical images are converted into mineable data by automated extraction of quantitative features that represent changes in radiographic enhancement patterns^7^. Using radiomic analysis on solid malignancies, imaging features can provide quantification of tumoral heterogeneity that is related to cell-density, necrosis, fibrosis and hemorrhage^8^. Enhancement patterns of CRLM on CT scans have been explored and show detectable differences in tumoral heterogeneity^9^. In addition, intrahepatic recurrence in the future liver remnant (FLR) is hypothesized to develop from occult metastases present at the time of resection but not detectable with conventional imaging^10,11^. Therefore, enhancement patterns of the hepatic parenchyma may be altered by underlying occult metastatic disease and can be quantified by image analysis^12^. These observations and preliminary results led our group to create a dataset to explore whether imaging features of the tumor and non-tumoral liver parenchyma are related to survival and hepatic disease-free survival following resection^13^.

**Fig. 1:**
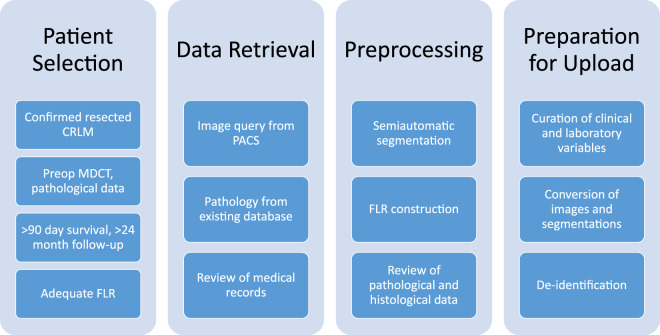
Overview of the data preparation workflow.

We are now releasing the dataset utilized for this project through The Cancer Imaging Archive (TCIA)^14^. It represents a large, single-institution consecutive series of patients with hepatic resection of CRLM and matched preoperative CT scans for quantitative image analysis. This is the same data used in the publication by Simpson *et al*.^13^ and represents the largest compilation of segmented, portal-venous, hepatic CT scans for image analysis of CRLM. The data collection and preparation workflow is summarized in Fig. 1. This is a step in the development of clinically useful imaging biomarkers for recurrence and survival. While the number of patients (n = 197) may not be large from a radiomics point of view, it is nonetheless the largest publicly available dataset of its kind at present. For a machine learning approach, a larger cohort would be desirable to support training and validation data splitting with sufficient sample sizes. Therefore, additional datasets are still required to refine and validate techniques and correlate imaging heterogeneity with underlying pathologic changes. Nonetheless, we hope that by releasing the data to the public, it can be useful to other researchers as part of a larger data set assembled from other public or private sources, or as an external validation set.

## Methods

### Patients

Approval from the Institutional Review Board of Memorial Sloan Kettering Cancer Center (MSKCC) was obtained for retrospective analysis with waiver of informed consent. This dataset includes patients (n = 197) from 384 consecutive hepatic resections previously utilized for two unrelated studies^15,16^. Inclusion criteria were (a) pathologically confirmed resected CRLM, (b) available data from pathologic analysis of the underlying non-tumoral liver parenchyma and hepatic tumor, (c) available preoperative conventional portal venous contrast-enhanced multi-detector computed tomography (MDCT) performed within 6 weeks of hepatic resection. Patients with 90-day mortality or that had less than 24 months of follow-up were excluded. Additionally, because pathologic and radiographic alterations of the non-tumoral liver parenchyma caused by hepatic artery infusion (HAI) of chemotherapy are not well described, any patient who received preoperative HAI was excluded. Finally, to obtain the most accurate FLR 3D-model, patients who underwent either local tumor ablation, more than 3 wedge resections in the FLR, or had no visible tumor on preoperative imaging were excluded.

### Clinical characteristics

Clinical, laboratory, or radiographic variables were collected from the electronic medical record and the Hepatopancreatobiliary Service prospectively-maintained database used for a previous study^15^. These included age, sex, lymph node status of primary, synchronous disease, number and size of hepatic lesions, extrahepatic disease, carcinoembryonic antigen (CEA) level, Clinical Risk Score, neoadjuvant chemotherapy, and hepatic artery infusion^17–19^.

Selected patients were also required to have pathologic re-review of the tumor and non-tumoral liver parenchyma^15,16^. As part of a previous study on the effects of chemotherapy on non-tumoral liver parenchyma, all resection specimens were reviewed for steatosis, sinusoidal dilation, and steatohepatitis^20,21^. Furthermore, all selected patients also had re-review of the dominant histologic response pattern of the tumor as part of a larger study to determine the pathologic alteration that drives the association between response and survival. Percentage mucin, fibrosis, and necrosis were reported for resected tumors by a blinded pathologist regardless of whether the patient received neoadjuvant chemotherapy^16^.

A summary of the demographic and clinicopathologic features of the selected patients is given in Table 1. The clinical and pathology variables are being made available in spreadsheet form on TCIA, for all 197 selected patients^22^. The full set of variables being released, and a description of their values and interpretation is given in Supplementary Table 1.

**Table 1:** Summary of the demographic, clinicopathologic, and outcome variables of the patient population (n = 197).

	Value
Age [years (range)]	61 (30–88)
Male sex	117 (59)
Major comorbidity	109 (55)
Body mass index [kg/m2 (range)]	26.8 (17.2–44.3)
Node-positive primary tumor [n (%)]	69 (35)
Synchronous CRLM [n (%)]	111 (56)
Multiple metastases [n (%)]	114 (58)
CRS (score 0–2)	117 (59)
CEA >200	3 (1.5)
Maximal tumor size [cm (mean ± SD)]	3.5 ± 2.6
Bilobar disease [n (%)]	86 (44)
Extrahepatic disease [n (%)]	17 (9)
Neoadjuvant chemotherapy [n (%)]	122 (62)
Preoperative PVE [n (%)]	23 (12)
Pathology of nontumoral liver [n (%)]	
Steatosis	68 (35)
Sinusoidal dilation	26 (13)
Steatohepatitis (grade C 4)	7 (4)
Pathology of index tumor	
Total response >75%	38 (19)
Tumor fibrosis >40%	24 (12)
Percentage necrosis [median (range)]	30 (0–90)
Percentage fibrosis [median (range)]	10 (0–100)
Percentage mucin [median (range)]	0 (0–100)

Data are expressed as n (%) unless otherwise specified. CRLM = colorectal liver metastases, CRS = clinical risk score, CEA = carcinoembryonic antigen, PVE = portal vein embolization, SD = standard deviation.

### CT Acquisition

**Fig. 2:**
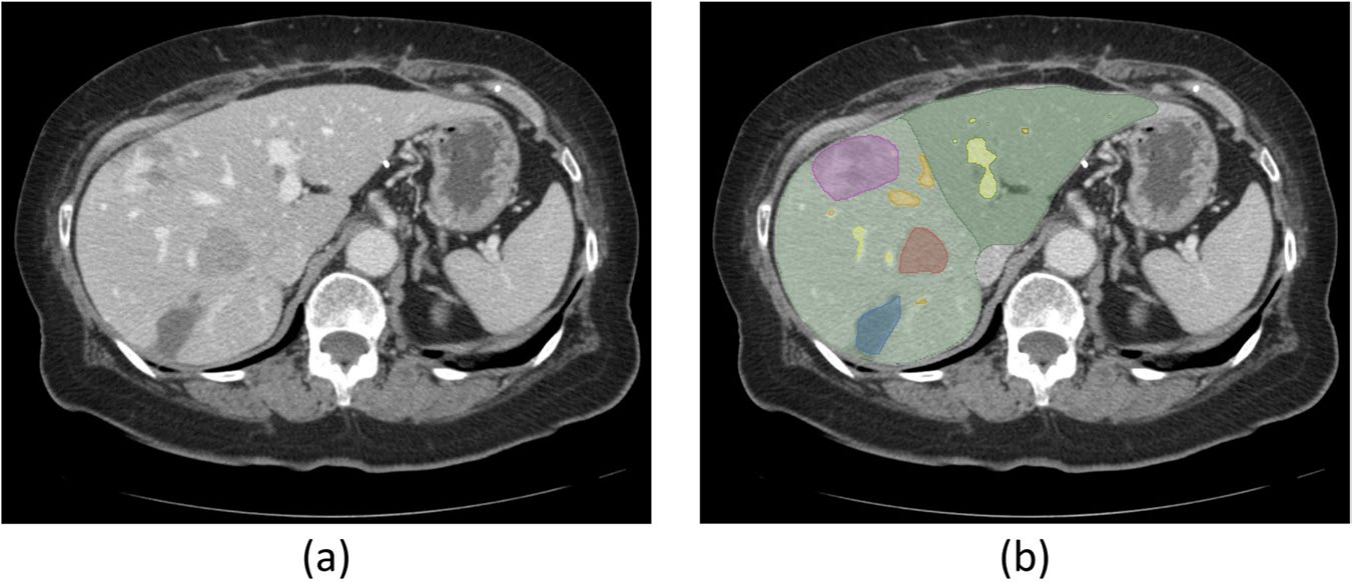
A sample CT image slice with segmentations. (**a**) A slice of the CT volume. (**b**) Segmentations of the liver (green), liver remnant (darker green), hepatic and portal veins (orange and yellow), and tumors (red, blue, purple).

Each patient included had a conventional portal venous phase contrast-enhanced CT scan within 6 weeks of surgery. Multidetector CT scanner (Lightspeed 16 and VCT, GE Healthcare, Wisconsin) was employed for abdominal imaging with main parameters: autoMA 220–380; noise index 12–14; rotation time 0.7–0.8 milliseconds; scan delay 80 seconds. In patients receiving neoadjuvant chemotherapy, the post-treatment/preoperative CT was used for image analysis. In patients undergoing a preoperative PVE, the pre-PVE CT was used for image analysis because PVE changes the appearance of parenchyma in CT imaging and the effect of PVE on results of hepatic enhancement patterns are unstudied. These preoperative CT scans are included in DICOM format for all 197 patients in the released dataset^22^. An example slice of an included CT scan can be seen in Fig. 2a.

### Image processing

Images were transferred from the picture archiving and communication system (PACS) to a workstation for image processing. Standard image processing techniques were used to segment the liver parenchyma from surrounding structures. Liver, tumors, vessels, and bile ducts were semi-automatically segmented and a 3D model was generated using Scout Liver (Pathfinder Technologies Inc., TN, USA). The performed liver resection was virtually drawn on the 3D model of the liver. Transection lines to generate the FLR were based on postoperative imaging and/or resection margin width from pathology analysis.

The masks corresponding to the segmentations of the liver, tumor(s), hepatic and portal vessels, along with the FLR were initially saved in the ITK MetaImage format (https://itk.org/Wiki/ITK/MetaIO), and converted to DICOM segmentation objects (DSO), in accordance with the Segmentation Information Object Definition, as specified in PS3.3: DICOM Information Object Definitions in the DICOM standard^23^, using the 3D Slicer python API (https://www.slicer.org)^24^. The following Slicer extensions were utilized during the conversion process: SlicerDevelopmentToolbox, DCMQI, PETDICOMExtension, QuantitativeReporting. Examples of the segmentation masks can be seen in Fig. 2b.

### Survival and recurrence data

Statistics for overall survival, disease-free survival, and hepatic disease-free survival were collected for all 197 patients. Columns indicating whether an event occurred, and time to event (or last follow up) are included in spreadsheet form alongside the clinical and pathological data on TCIA^22^. Hepatic disease-free survival events are defined as either recurrence inside the liver, or death. Disease-free survival events include any recurrence (inside or outside the liver) or death. At final follow up (median 102 months), 90 patients were alive, of which 75 had no evidence of hepatic recurrence, and 59 had no evidence of recurrence of any kind. The median time to event, computed via Kaplan-Meier analysis, was 76 months for overall survival, 53 months for hepatic disease-free survival, and 22 months for disease-free survival.

## Data Records

The preoperative portal venous contrast-enhanced CT scans for all 197 patients, along with corresponding segmentation masks of the liver, CRLM tumors, vessels, and FLR, are available on TCIA as collection “Preoperative CT and Recurrence for Patients Undergoing Resection of Colorectal Liver Metastases (Colorectal-Liver-Metastases)”^22^. These image and segmentation data are provided in de-identified DICOM format, where the segmentations of each patient are stored as separate masks in a unique DSO file per patient.

The corresponding clinical, pathology, and survival/recurrence variables for each patient are available as a single Microsoft Excel spreadsheet^22^. The first sheet contains the variable data for each patient, while the second contains a data dictionary describing each variable, which is reproduced in Supplementary Table 1. All subjects can be cross-referenced with their corresponding images and segmentation data via the “Patient-ID” variable, which corresponds to the subject’s DICOM patient ID. Survival time columns (overall_survival_months, months_to_DFS_progression, months_to_liver_DFS_progression) correspond to the time an event occurred, or, for censored observations, to the time of last follow up.

Note that, while this data set could be used for radiomic analysis, the data set as released does not include any extracted radiomic features. Instead, the images, segmentations, and clinicopathological variables are provided. Our rationale is that radiomic features vary depending on the software used to derive the features so we leave it to researchers to define their own methods.

## Technical Validation

### Patient selection

The included patients were a subset (n = 197) of 384 consecutive hepatic resections conducted at a single institution. The CT images represent standard of care for patients undergoing resection of CRLM. Patients were selected based on the needs of the dataset – in particular, based on confirmation of CRLM, availability of the relevant imaging and pathological data, with more than 24 months of follow up and at least 90-day survival. Patients that underwent major resections were selected as these patients are more likely to recur, and therefore have enough recurrence events for survival modeling. We excluded patients that underwent local tumor ablation, more than three wedge resections, or that had no visible tumor on the preoperative imaging, to ensure that the resulting FLR model would be as accurate as possible. Finally, patients that received preoperative HAI were excluded because one aim of this dataset is to facilitate study of imaging biomarkers that may exist in the non-tumoral liver parenchyma, and the effects of such treatment on the pathology and radiographic imaging of the liver parenchyma are not well understood. While similar concerns exist for patients in the cohort who received neoadjuvant chemotherapy, we note that the use of neoadjuvant chemotherapy is included in the clinicopathological variables provided with the data, and can therefore be used to control for these effects, or exclude such patients, as needed, by users of the data set.

### Clinical and pathology variables

The clinical and pathology variables for each patient were obtained from a prospectively maintained database that was used for a previous study^15^. All selected patients had their data supplemented with a review of medical records and pathologic re-review of the underlying non-tumoral liver parenchyma and hepatic tumor to gather various pathological and histological information, and the reported variables were based on standard scoring systems. In particular, non-alcoholic steatohepatitis was evaluated using the Kleiner-Brunt scoring system^20^. Within this scoring system, patients with a score of 1 or higher for steatosis, which indicates >5% parenchymal involvement in the histological evaluation, are indicated as having steatosis. Sinusoidal dilatation was evaluated using the Rubbia-Brandt grading system^21^, with scores of 1 or higher considered as indicative of sinusoidal injury. Clinical risk scores, which combine the presence of five factors associated with recurrence of CRLM after hepatic resection into a numeric score from 0 to 5, are also provided for 168 of the 197 patients^17^. Pathologic response, broken into three components as percentage mucin, percentage fibrosis, and percentage necrosis, was also evaluated by re-review of the histology slides for all patients by a pathologist blinded to the use of neoadjuvant chemotherapy^16^.

### Segmentations and future liver remnant

The segmentations of the liver, tumors, and vessels were produced semi-automatically using standard image processing and software (Scout Liver, Pathfinder Technologies Inc., TN, USA) and were conducted by an expert radiologist or fellow. Post-operative imaging and/or resection margin width was used to virtually draw the performed liver resection on the preoperative 3D model of the liver, to derive the FLR. The DICOM segmentation property codes for all segmentations were set based on standard SNOMED CT codes (https://www.snomed.org/). In particular, the FLR property code was assigned using a combination of SNOMED codes for “Liver” and “Residual”.

### De-identification

After patient selection, CT DICOM images were extracted from PACS and stored on a workstation for segmentation and processing. The images as well as resulting DSO segmentation files were de-identified to remove patient protected health information (PHI) using the TCIA de-identification process. This process utilizes the National Institute of Health (NIH) approved Clinical Trials Processor (CTP) to remove elements of PHI and ensures compliance with the US Health Insurance Portability and Accountability Act (HIPAA) and DICOM protocols. Certain private DICOM tags were also removed by updating the appropriate flag in the de-identification script to prevent accidental inclusion of PHI elements. Both MSKCC and TCIA teams conducted quality assurance on the final de-identified images prior to public release.

## Usage Notes

This dataset can be accessed through TCIA as collection “Preoperative CT and Recurrence for Patients Undergoing Resection of Colorectal Liver Metastases (Colorectal-Liver-Metastases)”^22^. All imaging and segmentation data are provided in standard DICOM format and can be viewed and converted using many publicly available open source tools, such as, for example, 3D Slicer (https://www.slicer.org/). Quantitative imaging features can be extracted using open-source libraries like pyradiomics (https://github.com/AIM-Harvard/pyradiomics), or directly in Slicer using the extension SlicerRadiomics.

### Supplementary information

Supplementary Table 1

## Data Availability

Code for converting DICOM images with segmentation masks to standard DICOM segmentation objects is available on GitHub: https://github.com/lassoan/LabelmapToDICOMSeg.
